# A near-zero quiescent power breeze wake-up anemometer based on a rolling-bearing triboelectric nanogenerator

**DOI:** 10.1038/s41378-024-00676-7

**Published:** 2024-04-08

**Authors:** Xianpeng Fu, Zhichao Jiang, Jie Cao, Zefang Dong, Guoxu Liu, Meiling Zhu, Chi Zhang

**Affiliations:** 1 CAS Center for Excellence in Nanoscience, Beijing Key Laboratory of Micro-nano Energy and Sensor, Beijing Institute of Nanoenergy and Nanosystems, Chinese Academy of Sciences, Beijing, 101400 China; 2 School of Nanoscience and Engineering, University of Chinese Academy of Sciences, Beijing, 100049 China; 3 Center on Nanoenergy Research, Guangxi Colleges and Universities Key Laboratory of Blue Energy and Systems Integration, School of Physical Science & Technology, Guangxi University, Nanning, 530004 China; 4 Institute of Intelligent Flexible Mechatronics, Jiangsu University, Zhenjiang, 212013 China; 5 College of Engineering, Mathematics and Physical Science, University of Exeter, Exeter, EX44QF UK

**Keywords:** Environmental, health and safety issues, Electrical and electronic engineering

## Abstract

Wind sensors have always played an irreplaceable role in environmental information monitoring and are expected to operate with lower power consumption to extend service lifetime. Here, we propose a breeze wake-up anemometer (B-WA) based on a rolling-bearing triboelectric nanogenerator (RB-TENG) with extremely low static power. The B-WA consists of two RB-TENGs, a self-waking-up module (SWM), a signal processing module (SPM), and a wireless transmission unit. The two RB-TENGs are employed for system activation and wind-speed sensing. Once the ambient wind-speed exceeds 2 m/s, the wake TENG (W-TENG) and the SWM can wake up the system within 0.96 s. At the same time, the SPM starts to calculate the signal frequency from the measured TENG (M-TENG) to monitor the wind speed with a sensitivity of 9.45 Hz/(m/s). After the wind stops, the SWM can switch off the B-WA within 0.52 s to decrease the system energy loss. In quiescent on-duty mode, the operating power of the B-WA is less than 30 nW, which can greatly extend the service lifetime of the B-WA. By integrating triboelectric devices and rolling bearings, this work has realized an ultralow quiescent power and self-waked-up wireless wind-speed monitoring system, which has foreseeable applications in remote weather monitoring, IoT nodes, and so on.

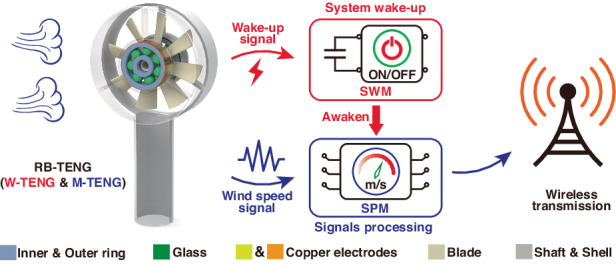

## Introduction

Wind power generation is playing an increasingly important role in the global power supply and contributing to reducing carbon emissions^1–3^. Anemometers^4,5^, as essential wind-speed monitoring devices, are distributed in every corner of the world for wind resource exploration and environmental monitoring^6–8^. By continuously receiving and analyzing wind-speed information from distributed wireless sensing nodes, meteorological departments can publish weather forecasts early to guide agricultural production and prevent natural disasters^9–12^. To date, widely distributed anemometers are powered mainly by batteries with limited working lifetimes and expensive maintenance costs^13–15^. To extend the service lifespan, the power consumption of anemometers needs to be further reduced.

Since they were invented in 2012, triboelectric nanogenerators (TENGs)^16,17^ have been widely used as environmental energy harvesters^18–20^ and active sensors^21–23^ because of their light weight^24^, high flexibility^25^, and plentiful material selection^26^. Currently, many TENG-based wind-speed monitoring sensors and integrated systems have been developed to prolong the driving time of supply batteries or eliminate batteries^27^. However, the reported TENG-based wind-speed sensors and systems always remain in high-power-consuming active mode during the entire operating process, resulting in considerable system energy waste^28^. To prolong the service lifetime, the power consumption of the system needs to be intelligently adjusted according to the working mode^26,29–31^. In 2015, “auto-wake-up technology” was reported for developing a near-zero quiescent power wireless sensing system with a long lifespan^32–37^. Hsieh et al. reported a low-power auto-wake-up image-reject receiver front-end^37^. The reported device is usually in low-power sleep mode and can be automatically awakened upon receiving a radio frequency (RF) signal. Zhang et al. reported an ultralow power disturbance detection system based on a biomimetic TENG, which can significantly prolong the battery life of an electronic system^38^. If the auto-wake-up technology can be applied to a TENG-based wind-speed monitoring system, then a wind-speed monitoring system with low quiescent power and a long lifespan may be realized.

Here, we propose a breeze wake-up anemometer (B-WA) based on a rolling-bearing TENG (RB-TENG) with extremely low static power. Two RB-TENGs are employed for system activation and wind-speed sensing. Once the ambient wind-speed exceeds 2 m/s, the wake TENG (W-TENG) and the self-waking module (SWM) can wake up the system within 0.96 s. At the same time, the signal processing module (SPM) starts to calculate the signal frequency from the measured TENG (M-TENG) to monitor the wind speed with a sensitivity of 9.45 Hz/(m/s). After the wind stops, the SWM can switch off the B-WA within 0.52 s to decrease the system energy loss. In the quiescent on-duty state, the operating power of the B-WA is less than 30 nW, which can greatly extend its service lifetime. By integrating triboelectric devices and rolling bearings, this work has realized an ultralow quiescent power and self-wake-up wireless wind-speed monitoring system, which has potential applications in remote weather monitoring, IoT nodes, and so on.

## Results and discussion

### Overview of the B-WA

**Fig. 1:**
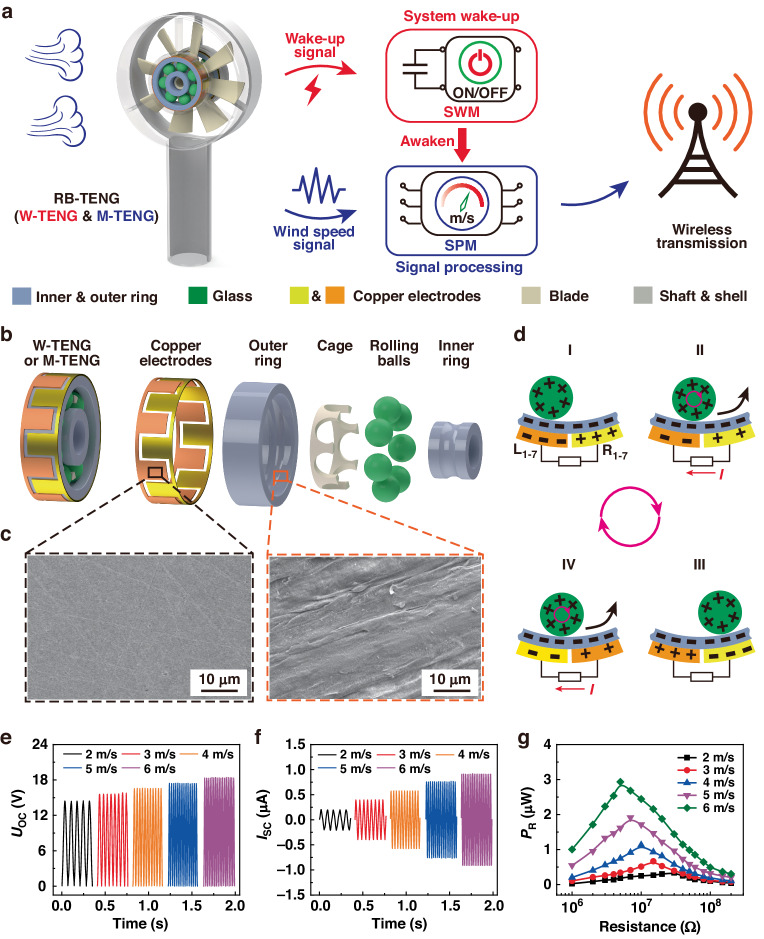
Overview of the breeze-awaken anemometer (B-WA) and the rolling-bearing triboelectric nanogenerator (RB-TENG). **a** Composition and workflow diagrams of the B-WA. The B-WA consists of two RB-TENGs, an SWM, an SPM, and a wireless transmitter. **b** Structural diagram of the RB-TENG. **c** SEM images showing the morphology of the copper electrode and the outer ring. **d** Working principle of the RB-TENG. **e** Output *U*_OC_ of the RB-TENG under various driving wind speeds. **f** Output *I*_SC_ of the RB-TENG under various driving wind speeds. **g** Instantaneous power output of the RB-TENG under various driving wind speeds

Figure 1 shows an overview of the ultralow static power B-WA and the basic electrical output performance of the RB-TENG. As indicated in Fig. 1a, the B-WA is composed of two RB-TENGs, an SWM, an SPM, and a wireless transmission unit. The two RB-TENGs are connected to the SWM and SPM for system activation and wind-speed sensing, respectively. For system activation, the W-TENG can generate electricity driven by the breeze and stored in the capacitor of the SWM. Subsequently, the SWM will activate the energy supply for the SPM when the stored voltage of the capacitor reaches the threshold voltage. Moreover, the M-TENG is utilized to transform the wind-speed information into detectable electrical signals. Then, the detectable electrical signals are modulated and analyzed by the SPM to derive the wind-speed information. Finally, the sensing data can be wirelessly transmitted over long distances between the B-WA and display terminals. Figure 1b shows the basic structure of the freestanding mode RB-TENG, which is prepared based on a rolling ball bearing. The RB-TENG consists of interdigital copper electrodes, an outer ring, a cage, rolling balls, and an inner ring. The interdigital copper electrodes are not in contact with the rolling balls to avoid affecting the normal rotation of the bearings. The fabrication process of the RB-TENG is described in detail in the “Experimental section”.

Figure 1c shows morphology images of the copper electrode and the inner surface of the outer ring obtained by scanning electron microscopy (SEM). The outer ring is fabricated with polyformaldehyde (POM) materials, which indicates excellent wear resistance. The rolling-induced charge transfer process of the RB-TENG is depicted in Fig. 1d. After a period of rolling friction between the POM outer ring and glass rolling balls, equal quantities of opposite charges are generated on the rolling balls and outer ring surfaces with different electronegativities. In the initial state, the rolling balls are located at the matching position with electrode groups L_1-7_, as depicted in Fig. 1d (I). There is no current passing through the external load in this state. When the rolling-bearing is driven to rotate by the blade, the glass balls will gradually roll from electrode groups L_1-7_ to electrode groups R_1-7_, as indicated in Fig. 1d (II). Driven by the potential difference between the two electrode groups, the forward current from electrode groups R_1-7_ to electrode groups L_1-7_ is generated. Until the balls reach the position completely facing electrode groups R_1-7_, as depicted in Fig. 1d (III), the electrons will move from electrode groups L_1-7_ to electrode groups R_1-7_ and neutralize the positive charges. When the balls continuously roll counterclockwise to electrode groups L_1-7_, the reverse current flows from electrode groups L_1-7_ to electrode groups R_1-7_ through an external load with a nonelectrostatic equilibrium state between the two electrode groups, as depicted in Fig. 1d (IV). Until the balls return to the position completely facing electrode groups L_1-7_, all the electrons on electrode groups R_1-7_ flow back to electrode groups L_1-7_ and establish a new electrostatic balance, as shown in Fig. 1d (I). The RB-TENG generates an alternating current (AC) signal.

The output performance of the RB-TENG is measured in detail by an air blower providing driving wind at different speeds. Figure 1e depicts the signal waveforms of the open-circuit voltage (*U*_OC_) under various driving wind speeds. The output *U*_OC_ of the RB-TENG slightly increases with increasing wind speed. As depicted in Fig. S1, the maximum value of the *U*_OC_ increases from 14.41 V to 18.41 V as the wind-speed increases from 2 to 6 m/s. The probable reason is that the distance between the electrodes and rolling balls decreases with increasing centrifugal force. Figure 1f shows the signal waveforms of the short-circuit current (*I*_SC_) under various driving wind speeds. The output *I*_SC_ increases with increasing driving wind speed. As indicated in Fig. S2, the peak value of the *I*_SC_ increases from 0.2 μA to 0.91 μA as the driving speed increases from 2 to 6 m/s. The impedance characteristics of the RB-TENG were measured in detail under various wind speeds, as shown in Fig. 1g. The calculated instantaneous power at each wind-speed first increases with increasing external resistance and then decreases. The optimum output power increases with increasing wind speed. As shown in Fig. S3, the optimum output power increases from 0.33 to 2.93 μW as the wind-speed increases from 2 to 6 m/s.

### Awakening characteristics of the SWM

**Fig. 2:**
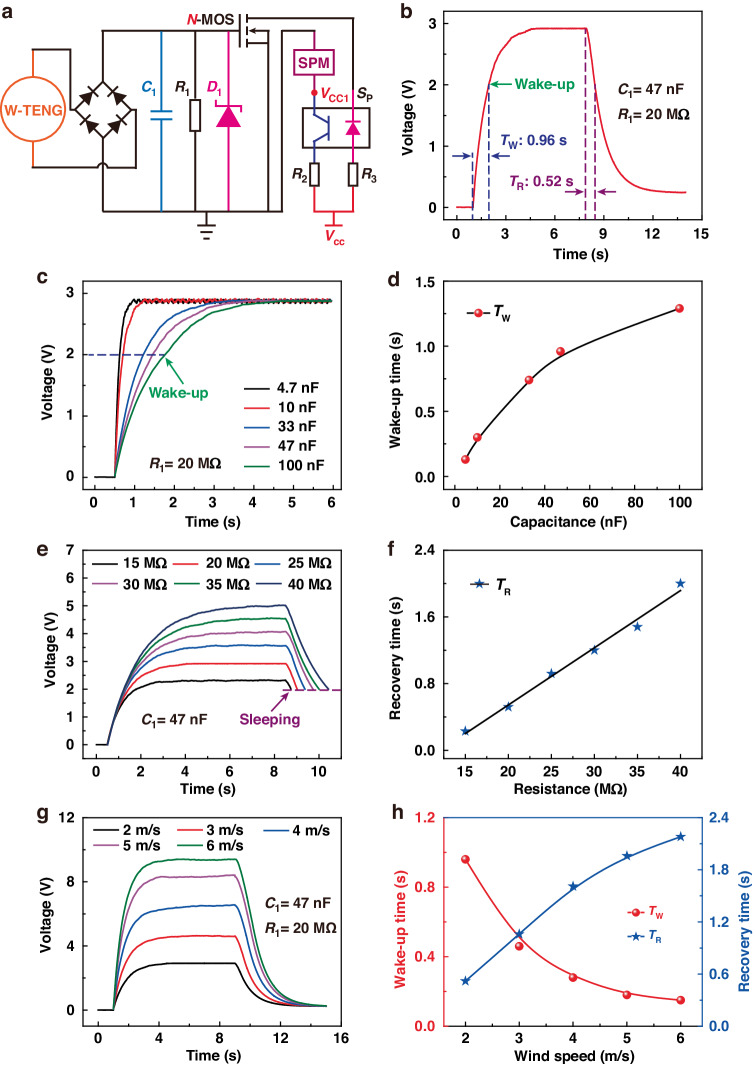
Circuit diagram and awakening characteristics of the SWM. **a** Equivalent circuit of the SWM. **b** The voltage waveform of the store capacitor *C*_1_ at 2 m/s. **c** Charging curves of *C*_1_ at 2 m/s. **d** Relationship between the wake-up time and capacitance *C*_1_. **e** Voltage waveforms of *C*_1_ under various resistances *R*_1_. **f** Relationship between the recovery time and the resistance of *R*_1_. **g** Voltage waveforms of *C*_1_ at different wind speeds. **h** Wake-up and recovery times with different wind speeds

Figure 2 depicts the circuit diagram and the working characteristics of the SWM. One of the RB-TENGs (W-TENG) is connected to the SWM for system activation. As shown in Fig. 2a, the SWM is composed of a full-bridge rectifier, storage capacitor *C*_1_, Zener diode *D*_1_, N-MOSFET (N-MOS), optocoupler switch *S*_P_ and resistors *R*_1_-*R*_3_. The resistor *R*_1_ is employed to provide a discharge circuit for the capacitor *C*_1_ and prevent the B-WA from being accidentally awakened. Zener diode *D*_1_ is used to provide overvoltage protection for the N-MOS. Similarly, resistors *R*_2_ and *R*_3_ are mainly used to limit the current passing through the optocoupler switch *S*_P_. Driven by a breeze, the electrical output from the W-TENG is first rectified through the full-bridge rectifier and then charged for capacitor *C*_1_. Under continuous charging by the W-TENG, the voltage of capacitor *C*_1_ gradually increases. Once the storage voltage of *C*_1_ reaches the turn-on voltage, the N-MOS is switched on in the low-on-resistance state. Then, the SPM can be activated and operated in active mode with the turn-on *S*_P_. At the same time, the SPM starts to analyze the signal frequency from the M-TENG and wirelessly transmits the monitoring wind-speed information via the wireless transmitter. After the wind stops, the storage voltage of *C*_1_ continuously decreases due to the energy consumption of resistor *R*_1_. Until the storage voltage decreases to the gate threshold voltage of the N-MOS, the N-MOS is switched off, and the SPM switches to sleep mode with the turn-off *S*_P_ to reduce power consumption.

Figure 2b depicts the voltage variation curve of the store capacitor *C*_1_ = 47 nF under R_1_ = 20 MΩ. Driven by a light breeze of 2 m/s, the storage voltage can rise from 0 V to the threshold voltage of 2 V within 0.96 s, and the system will be awakened immediately. Under the continuous energy input from the W-TENG and the energy consumption of resistor *R*_1_, the voltage of capacitor *C*_1_ can remain at 2.92 V after the energy balance. When the driving wind is stopped at this time, the storage voltage will drop from 2.92 V to 2 V within 0.52 s, and the system can be immediately turned off. The wake-up time (*T*_W_) and recovery time (*T*_R_) are two key parameters for the SWM and are determined by the rate of increase and decrease, respectively, of the storage voltage. The dependence of the storage voltage of capacitor *C*_1_ on the different values of *C*_1_ and *R*_1_ are investigated systematically. Figure 2c shows the charging curves of *C*_1_ with different capacitances under a driving wind speed of 2 m/s. The rate of increase in the storage voltage decreases with increasing capacitance. The saturation voltages with different capacitances *C*_1_ are maintained at approximately *R*_1_ = 20 MΩ. The *T*_W_ increases with increasing capacitance *C*_1_. When the capacitance *C*_1_ increases from 4.7 nF to 100 nF, the *T*_W_ increases from 0.13 s to 1.29 s, as depicted in Fig. 2d. Figure 2e shows the voltage waveforms of capacitor *C*_1_ for various *R*_1_ values. The saturation voltage of capacitor *C*_1_ increases with increasing resistance *R*_1_. As indicated in Fig. 2f, the *T*_R_ increases with increasing resistance *R*_1_. When *R*_1_ increases from 15 MΩ to 40 MΩ, the *T*_R_ increases from 0.23 s to 2 s. In general, the SWM is expected to have a smaller capacitance and resistance from the perspective of faster wake-up and recovery times. Nevertheless, the system may be mistakenly awakened by sudden and intense environmental stimuli with too little capacitance or may remain unable to awaken at all times with too little resistance. To avoid erroneous wake-up and maintain a fast response simultaneously, the SWM is designed with *C*_1_ = 47 nF and *R*_1_ = 20 MΩ. Figure 2g shows the voltage curves of *C*_1_ with various driving wind speeds. Both the rising rate and saturation value of the storage voltage increase with increasing driving wind. The *T*_W_ of the SWM decreases with increasing wind speed, while that of the *T*_R_ decreases, as shown in Fig. 2h. The *T*_W_ of the SWM decreases from 0.96 s to 0.15 s, and the *T*_R_ increases from 0.52 s to 2.18 s within the range of 2 m/s to 6 m/s.

### Wind speed sensing characteristics of the B-WA

**Fig. 3:**
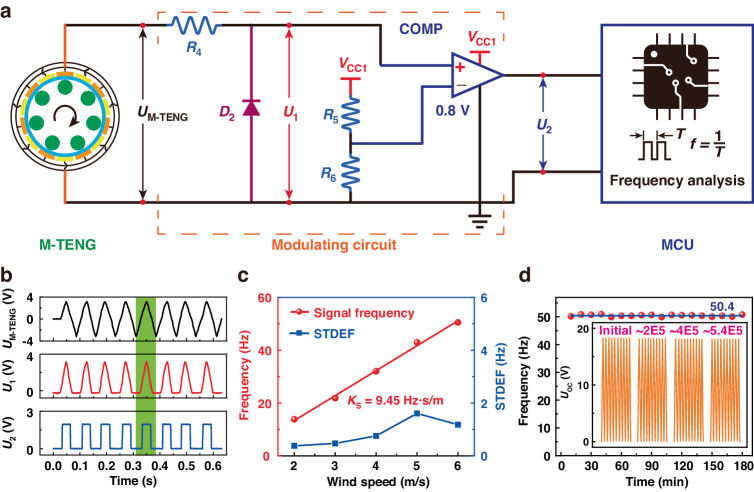
Wind speed sensing principle and performance of the B-WA. **a** Working principle of the SPM. **b** Output voltage signal waveform of the M-TENG, diode, and comparator. **c** The measurement frequency and calculated standard deviation under various wind speeds. **d** Output steadiness of the B-WA. The inset indicates the *U*_OC_ of the RB-TENG during 5.4 × 10^5^ cycles

The wind-speed sensing principle and performance of the B-WA are illustrated in Fig. 3. By calculating the frequency of the M-TENG output electrical signal, the SPM can be used to obtain the wind speed. Figure 3a depicts the working principle of the SPM. The SPM consists of a modulating circuit and a single-chip computer (MCU). The modulating circuit consists of three resistors *R*_4_, *R*_5_, and *R*_6_, a rectifier diode *D*_2_, and a comparer. The output AC electrical signal of the M-TENG is first modulated to a detectable level signal by the modulating circuit. The MCU can analyze the level signal from the modulating circuit and obtain the wind-speed information. The reference voltage of the comparer is 0.8 V provided by a 3.7 V power supply with resistors *R*_5_ and *R*_6_. Driven by the breeze, as depicted in Fig. 3b, the output AC electrical signals *U*_M-TENG_ are transformed into DC electrical signals *U*_1_ with the same frequency by the rectifier diode *D*_2_. If the voltage *U*_1_ is below the reference voltage of 0.8 V, then the comparer outputs a low-level signal. Once the voltage *U*_1_ exceeds 0.8 V, the comparer outputs the corresponding high-level signal. The MCU can calculate the monitoring information according to the level signal *U*_2_ output by the comparer. Figure 3b compares and summarizes the signal waveforms of the *U*_M-TENG_, *U*_1_, and *U*_2_, and their frequencies are basically unchanged after the signal is modulated. Figure 3c shows the frequency measured by the MCU and the calculated standard deviation under the various driving wind speeds. The measured frequency increases linearly with increasing driving wind speed. The measured frequency increases from 13.85 to 50.51 Hz as the driving wind increases from 2 m/s to 6 m/s. The monitoring sensitivity of the B-WA can reach 9.45 Hz/(m/s), which shows excellent performance for wind-speed sensing. According to 20 repeated measurements at each data point, the standard deviation is less than 1.6 Hz. The B-WA also demonstrated excellent sensing accuracy. The standard deviation of the wind-speed measurements may mainly be caused by the slight variation in the distance between the glass rolling ball and electrode during the rotation process. Moreover, the stability of the B-WA for wind-speed sensing is also studied. As indicated in Fig. 3d, the output voltage waveform of the M-TENG can always remain unchanged, which is a prerequisite for maintaining the accuracy of wind-speed sensing. During the 5.4 × 10^5^ measurement cycles, the calculated frequency of the MCU can stabilize at 50.4 Hz (5.92 m/s) at 6 m/s. The B-WA exhibits excellent stability for wind-speed sensing.

### Application demonstration of the B-WA

**Fig. 4:**
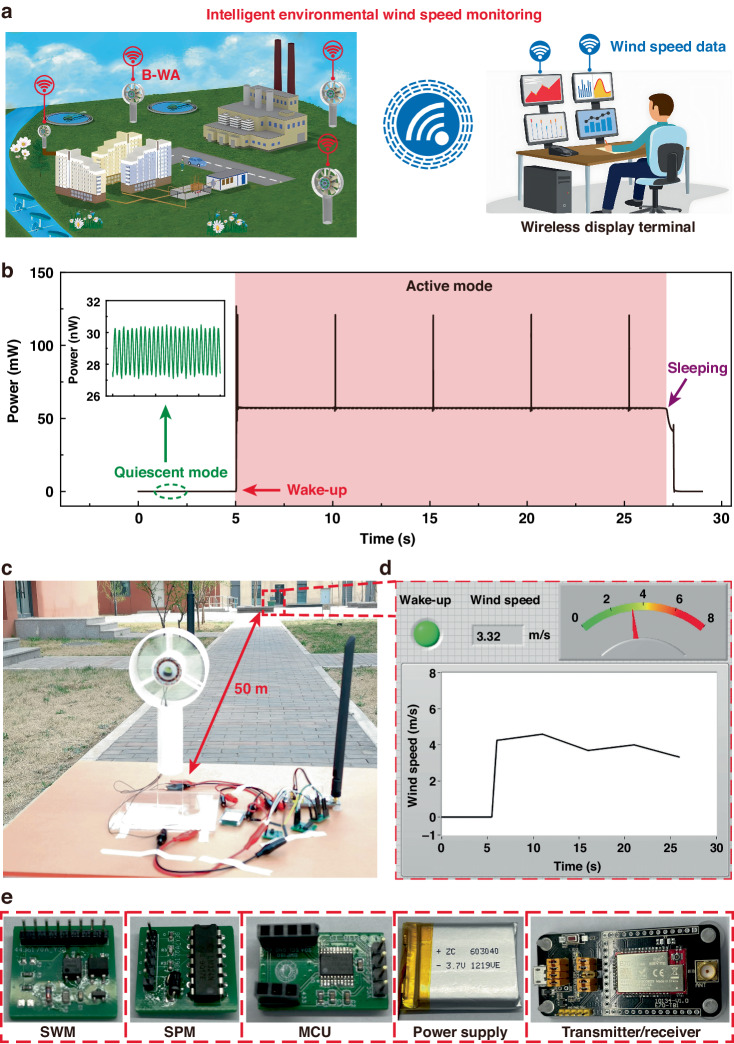
Demonstration of the B-WA. **a** Application prospects of the B-WA in intelligent environmental wind-speed monitoring. **b** Dynamic power consumption variation in the B-WA in the quiescent state and the active state. **c** Photo of the B-WA in an outdoor environment. **d** Display interface on the laptop. **e** Photos of the SWM, SPM, MCU, power supply, and wireless transmitter/receiver

Figure 4 demonstrates the potential applications of the B-WA in wind-speed monitoring. As depicted in Fig. 4a, the B-WA can realize long-term ambient wind-speed monitoring with near-zero on-duty power distributed in the natural environment. Combined with wireless communication technology, the monitoring wind-speed data can be wirelessly transmitted and displayed at the remote display terminal. Figure 4b shows the dynamic power consumption of the B-WA during the quiescent state and the working state. In the initial state, the B-WA is operated in quiescent mode. There is only a minute current through the opened switch *S*_P_ and cutoff N-MOS in the system. The static power is less than 30 nW, as shown in the illustration of Fig. 4b. Once the B-WA is woken up by the exciting wind, the system immediately enters the active state for information collection and wireless data transmission. The operating power of the system increases from 30 nW to 121 mW. After the driving wind stops, the system can autonomously return to the quiescent mode to reduce power consumption. In the active state, the B-WA can transmit collected wind-speed information every 5 s. The frequency of data transmission can be adjusted in the program. The working stage of the B-WA in the active state can be divided into a waiting stage and a data transmission stage. The power consumption of the B-WA is different in these two stages. In the wireless data transmission stage, the power consumption of the B-WA increases from 56.9 mW to 121 mW. The power consumption of the wireless transmitter correspondingly increases from 32.5 mW to 84.2 mW, as shown in Fig. S4. Figure 4c demonstrates the practical application of B-WA in natural outdoor environments for wind-speed monitoring. A receiver is connected to a laptop and positioned 50 m from the B-WA as the wireless receiving terminal to receive and display the wind-speed information. Figure 4d shows the display interface on the laptop, which includes a wake-up indicator, real-time measurements, and historical wind-speed data. Figure 4e shows photos of the SWM, SPM, MCU, power supply, and wireless transmitter/receiver. With its ultralow quiescent power, the B-WA can provide wind-speed information over a long service lifetime, which has foreseeable applications in remote weather monitoring, IoT nodes, and so on.

## Conclusions

In summary, we propose a near-zero quiescent power B-WA based on an RB-TENG for long-term wind-speed information monitoring. Two RB-TENGs are employed for system activation and wind-speed sensing. Once the ambient wind-speed exceeds 2 m/s, the W-TENG and the SWM can wake up the system within 0.96 s. At the same time, the SPM starts to calculate the signal frequency from the M-TENG to monitor the wind speed with an excellent sensitivity of 9.45 Hz/(m/s). The standard deviation of the B-WA is less than 1.6 Hz, which indicates excellent sensing accuracy. After the wind stops, the SWM can switch off the B-WA within 0.52 s to lower the energy consumption. In quiescent on-duty mode, the operating power of the B-WA is less than 30 nW, which can greatly extend the service lifetime of the B-WA. By integrating the triboelectric devices and rolling bearings, in this work, an ultralow quiescent power and self-wake-up wireless wind-speed monitoring system is realized, which has potential applications in remote weather monitoring, IoT nodes, and so on.

### Experimental section

#### Preparation of the RB-TENG

A commercial rolling-bearing is chosen as the substrate of the RB-TENG. The inner and outer rings are fabricated from the super wear-resistant POM material, while the material of the ball is glass. The external diameter, internal diameter, and height are 35 mm, 10 mm, and 11 mm, respectively. Fourteen pieces of copper film are cut from a copper roll as the electrodes. The size of each copper film is 11 × 6.8 × 0.06 mm. The copper films are coated at the external wall of the rolling ball bearing to form the two electrode groups. The copper films are welded to the enameled copper wire. The spacing between adjacent copper films is ~1 mm.

#### Preparation of the shell and blade

Both the shell and blade are fabricated with 3D printing technology. Two pieces of shell are processed separately with 8200 Pro resin material. The size of the shell is 170 × 89 × 38 mm. The blade is printed with 8228 resin material. The maximum tip distance of the blade is 75 mm. The maximum width of the blade is 27.5 mm.

#### Measurement

An air blower is used to offer a speed-adjustable driving wind for the B-WA. The display interface in the wireless terminal is designed with LabVIEW software. The model information of the components in the SWM includes N-MOS (2N7002) and an optocoupler switch (AB45S). The SPM consists of three resistors, a rectifier diode *D*_2_ (1N4001), a comparer (LM324N), and an MCU (STM32L031f6p6). The model of the wireless transmitter/receiver is CC1310.

### Supplementary information

Revised Supporting Information
