# Supplementary material for: A near-zero quiescent power breeze wake-up anemometer based on a rolling-bearing triboelectric nanogenerator
Source: Microsyst Nanoeng. 2024 Apr 8;10:51. doi: 10.1038/s41378-024-00676-7 (PMC11002024; doi:10.1038/s41378-024-00676-7)
Supplement: Supplementary file 1 — Revised Supporting Information [file 41378_2024_676_MOESM1_ESM.docx]

**Supporting Information**

**A** **Near-Zero** **Quiescent Power** **Breeze Wake-up** **Anemometer Based on Rolling Bearing Triboelectric Nanogenerator**

Xianpeng Fu^1, 2, #^, Zhichao Jiang^1, 3, #^, Jie Cao^1, 4^, Zefang Dong^1, 2^, Guoxu Liu^1^, Meiling Zhu^5^, and Chi Zhang^1, 2, 3, *^

^1^ CAS Center for Excellence in Nanoscience, Beijing Key Laboratory of Micro-nano Energy and Sensor, Beijing Institute of Nanoenergy and Nanosystems, Chinese Academy of Sciences, Beijing 101400, China

^2^ School of Nanoscience and Engineering, University of Chinese Academy of Sciences, Beijing 100049, China

^3^ Center on Nanoenergy Research, School of Physical Science and Technology, Guangxi University, Nanning 530004, China

^4^ Institute of Intelligent Flexible Mechatronics, Jiangsu University, Zhenjiang 212013, China

^5^ College of Engineering, Mathematics and Physical Science, University of Exeter, Exeter EX4 4QF, UK

^#^ These authors contributed equally: Xianpeng Fu, Zhichao Jiang

^*^Corresponding author: C. Zhang (czhang@binn.cas.cn).


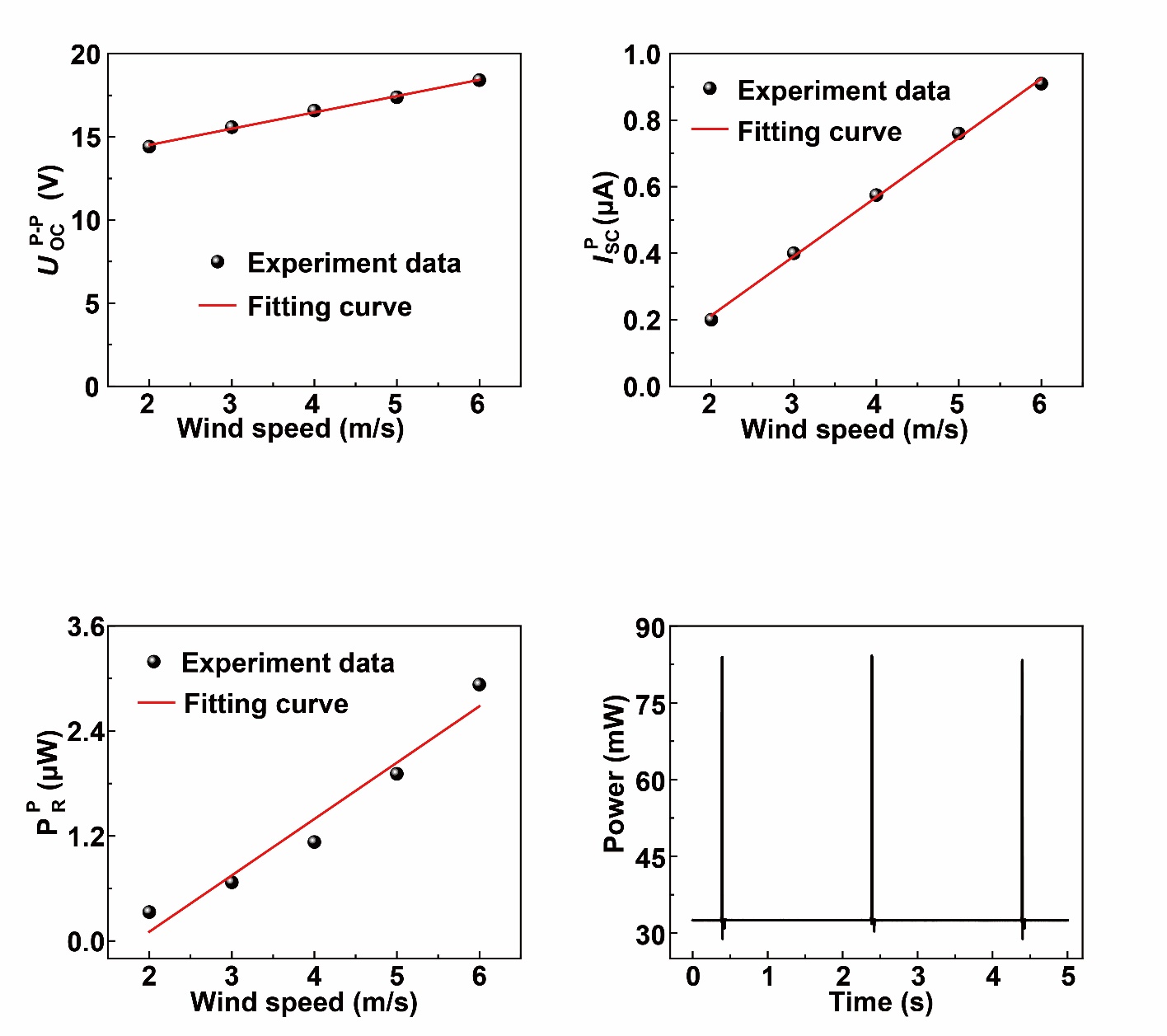


**Fig. S1. Variation of *U*_OC_ with the enlarging wind speed.**


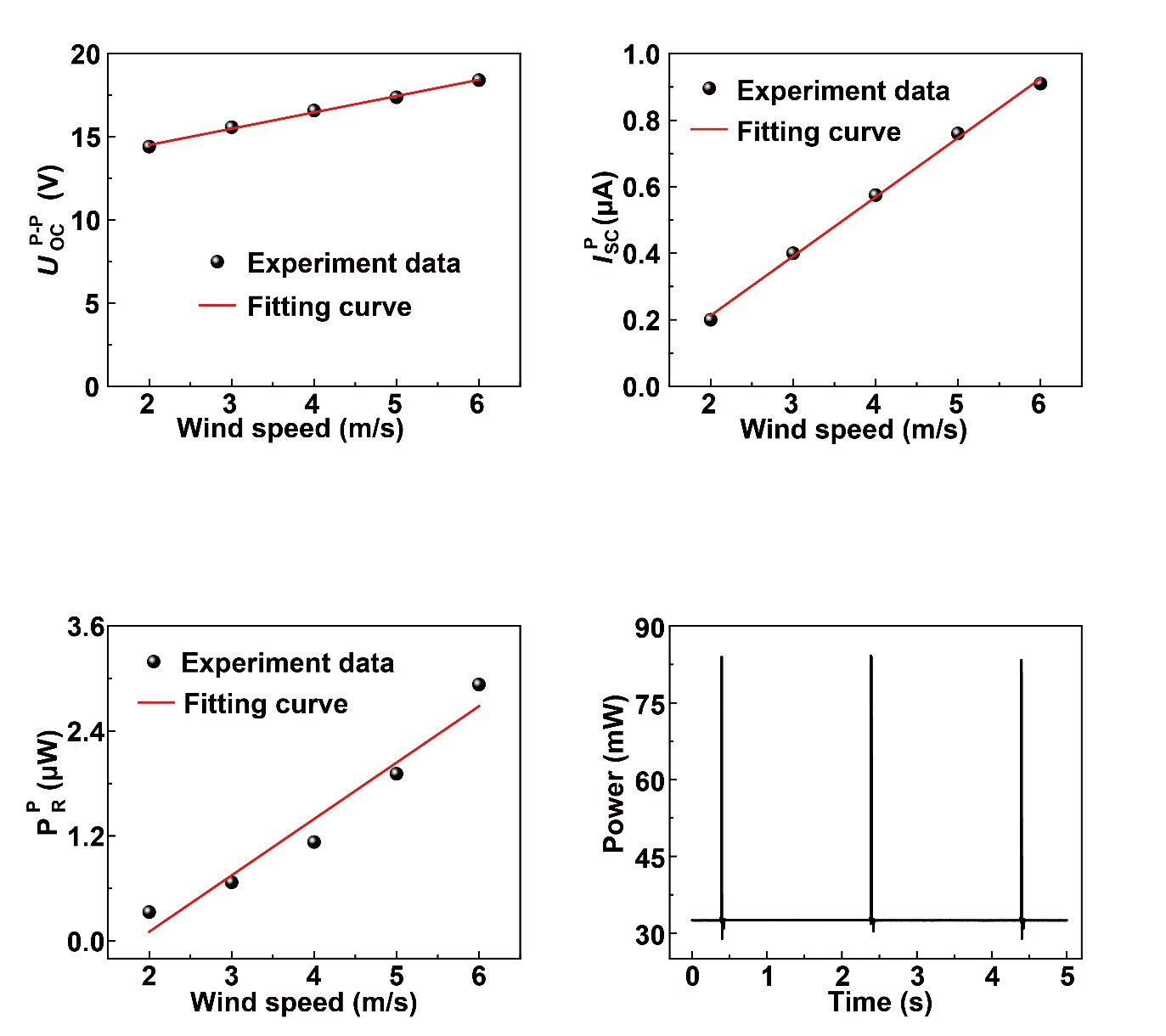


**Fig. S2. Variation of *I*_SC_ with the enlarging wind speed.**


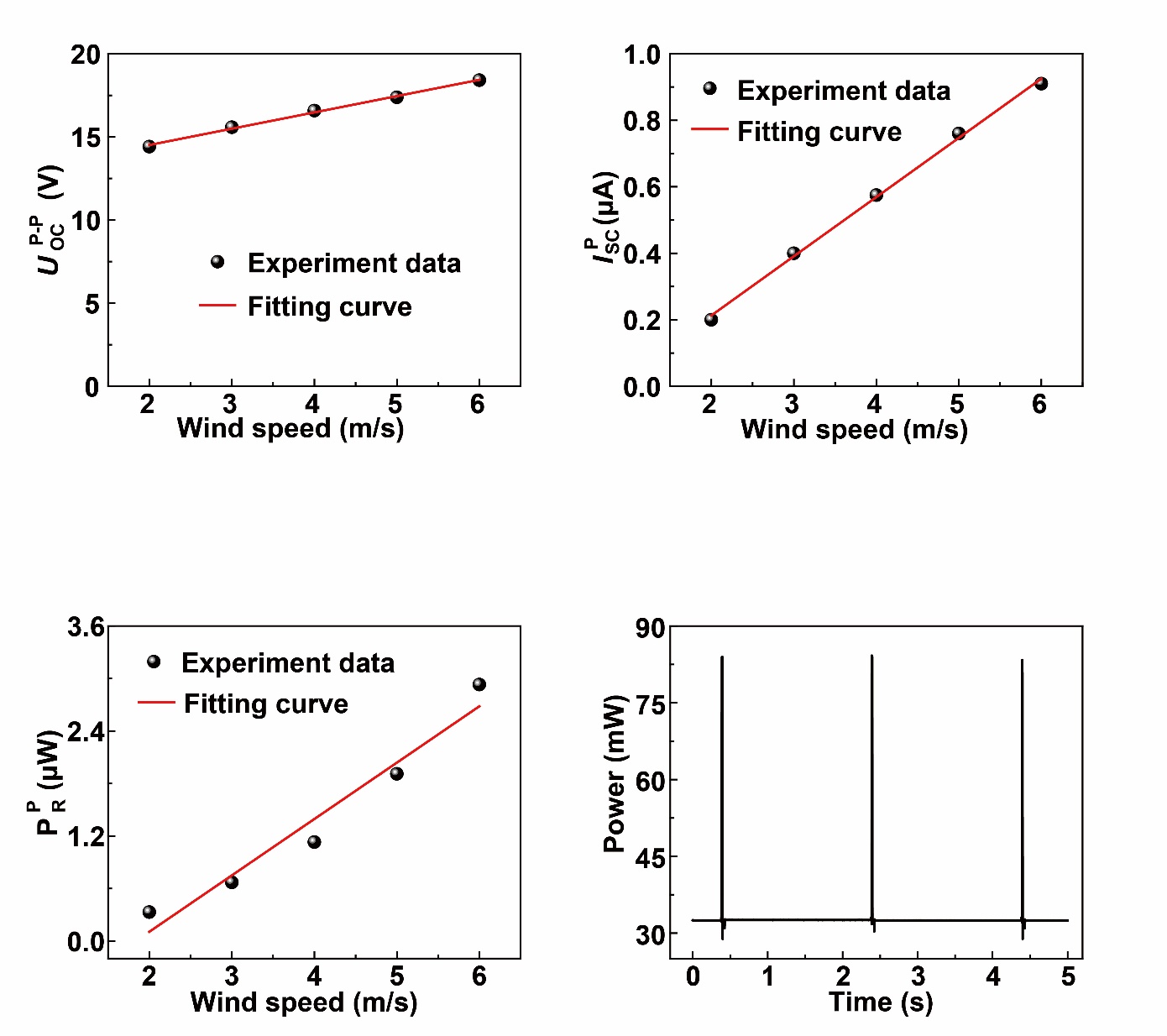


**Fig. S3. Variation of the maximum instantaneous power with the enlarging wind speed.**


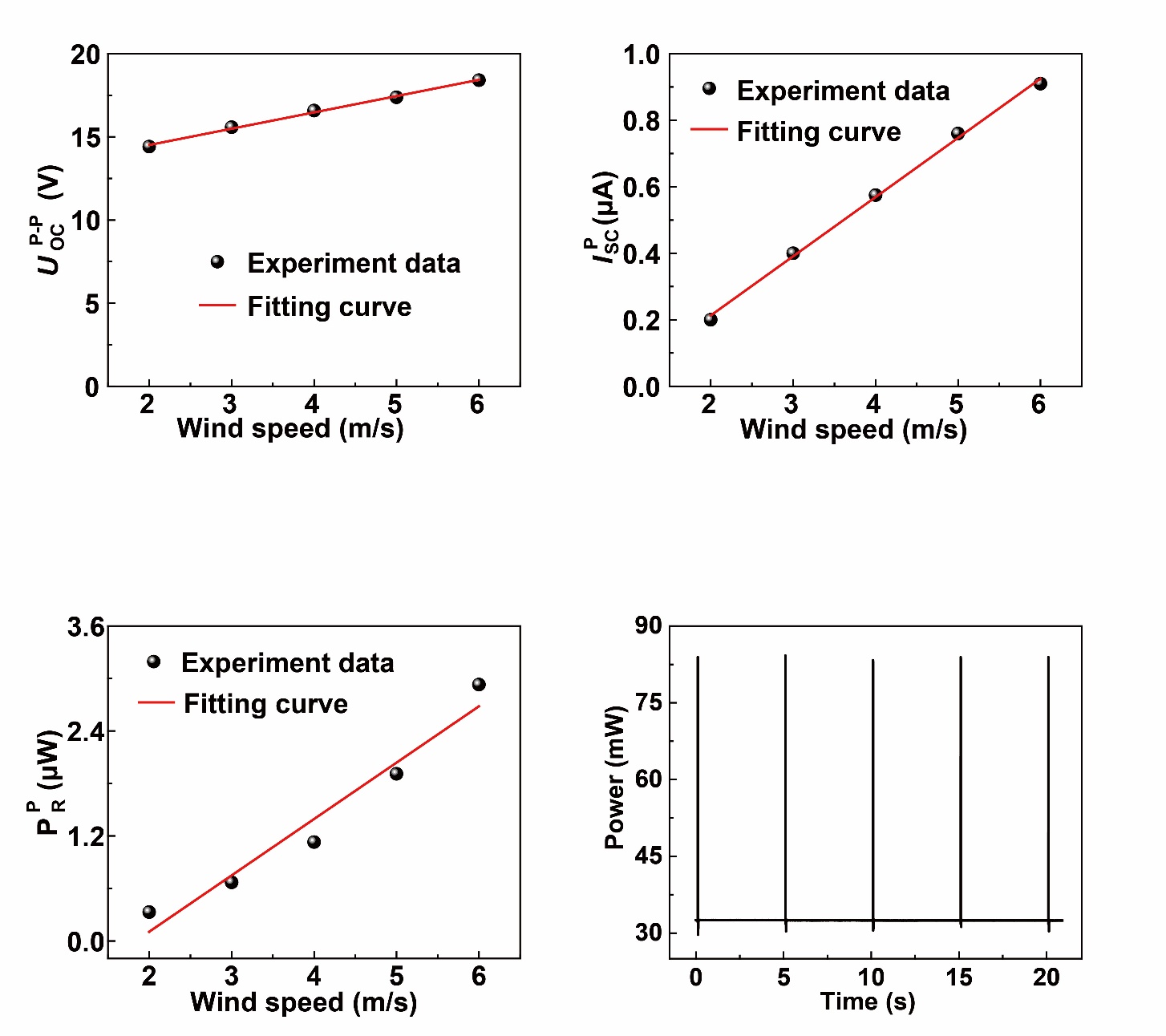


**Fig. S4. Operating Power of the wireless transmitter.**
